# A Fish Galectin-8 Possesses Direct Bactericidal Activity

**DOI:** 10.3390/ijms22010376

**Published:** 2020-12-31

**Authors:** Tengfei Zhang, Shuai Jiang, Li Sun

**Affiliations:** 1 CAS Key Laboratory of Experimental Marine Biology, CAS Center for Ocean Mega-Science, Institute of Oceanology, Chinese Academy of Sciences, 7 Nanhai Road, Qingdao 266071, China; zhangtengfei18@mails.ucas.ac.cn; 2 Laboratory for Marine Biology and Biotechnology, Qingdao National Laboratory for Marine Science and Technology, 1 Wenhai Road, Qingdao 266237, China; 3 University of Chinese Academy of Sciences, 19 Yuquan Road, Beijing 100049, China

**Keywords:** galectin-8, *Cynoglossus semilaevis*, bacterial binding, bactericidal, immune defense

## Abstract

Galectins are a family of animal lectins with high affinity for β-galactosides. Galectins are able to bind to bacteria, and a few mammalian galectins are known to kill the bound bacteria. In fish, no galectins with direct bactericidal effect have been reported. In the present study, we identified and characterized a tandem repeat galectin-8 from tongue sole *Cynoglossus semilaevis* (designated CsGal-8). CsGal-8 possesses conserved carbohydrate recognition domains (CRDs), as well as the conserved HXNPR and WGXEE motifs that are critical for carbohydrate binding. CsGal-8 was constitutively expressed in nine tissues of tongue sole and up-regulated in kidney, spleen, and blood by bacterial challenge. When expressed in HeLa cells, CsGal-8 protein was detected both in the cytoplasm and in the micro-vesicles secreted from the cells. Recombinant CsGal-8 (rCsGal-8) bound to lactose and other carbohydrates in a dose dependent manner. rCsGal-8 bound to a wide range of gram-positive and gram-negative bacteria and was co-localized with the bound bacteria in animal cells. Lactose, fructose, galactose, and trehalose effectively blocked the interactions between rCsGal-8 and different bacteria. Furthermore, rCsGal-8 exerted potent bactericidal activity against some gram-negative bacterial pathogens by directly damaging the membrane and structure of the pathogens. Taken together, these results indicate that CsGal-8 likely plays an important role in the immune defense against some bacterial pathogens by direct bacterial interaction and killing.

## 1. Introduction

Galectins are a family of animal lectins that bind β-galactosides through conserved carbohydrate recognition domains (CRDs) [1]. Galectins are evolutionarily conserved and widely present in a variety of species, from primitive to advanced organisms [2,3]. In mammals, galectins have been identified and classified into three types: the proto type that contains only one CRD, the tandem repeat type that contains two CRDs connected by a linker peptide, and the chimera type that contains one CRD and a non-lectin domain [4,5,6]. Galectin-8 (Gal-8) belongs to the tandem repeat type galectins comprised of two CRDs [7,8].

The innate immune system is the first line of defense against pathogen infection. Recognition of pathogen and non-self in innate immune system is mediated by pattern recognition receptors (PRRs). Reports have demonstrated that galectins bind the glycoconjugates on the surface of invasive microbes (virus, bacteria, fungi and parasites) and function as PRRs in innate immunity [9]. For example, human Gal-3 bound *Neisseria gonorrhoeae* through lipooligosaccharides [10], and hamster Gal-3 bound *Schistosoma mansoni* through LacdiNAc-Glycans [11]. In fish, galectins have been demonstrated to participate in pathogen recognition as well. Gal-1, a proto type galectin, from rock bream (*Oplegnathus fasciatus*) bound and agglutinated a wide range of bacteria [12], and the Gal-1 of zebrafish bound infectious hematopoietic necrosis virus (IHNV) in a carbohydrate-dependent manner [13]. The tandem-repeat type Gal-9 of large yellow croaker (*Larimichthys crocea*) and yellow catfish (*Pelteobagrus fulvidraco*) also showed bacterial binding and agglutinating abilities [14,15]. 

Recently, evidences have indicated that some mammalian galectins are able to kill pathogens directly. For example, human Gal-3 binds to *Candida albicans* expressing β-1,2-linked oligomannans and induces death of *Candida albicans* [16]. Reports of fish galectins with bacteriostatic or bactericidal effects are rare. AJL-1, a proto type galectin of Japanese eel (*Anguilla japonica*), was reported to inhibit the biofilm formation of *Aggregatibacter actinomycetemcomitans* [17]. The galectin-8 and -9 of mandarin fish (*Siniperca chuatsi*) were shown to inhibit the growth of some bacterial pathogens [18]. Another study showed that the chemically synthesized dimerization region of *Channa striatus* Gal-4, when tagged to pentamer oligotryptophan, exhibited bactericidal activity against *Vibrio harveyi* [19]. To our knowledge, no fish galectin with direct bacterial killing capacity has been documented. 

In this study, we identified a tandem repeat type galectin (named CsGal-8) from tongue sole *Cynoglossus semilaevis*, an important species of aquaculture fish. We examined the carbohydrate and bacterial binding activities of recombinant CsGal-8 (rCsGal-8), and investigated the bactericidal effect of rCsGal-8 on its target bacteria. Our results indicated that CsGal-8 is a broad-range bacteria binding lectin with strong and direct killing effect on some gram-negative bacteria.

## 2. Results

### 2.1. CsGal-8 Sequence Analysis

**Figure 1:**
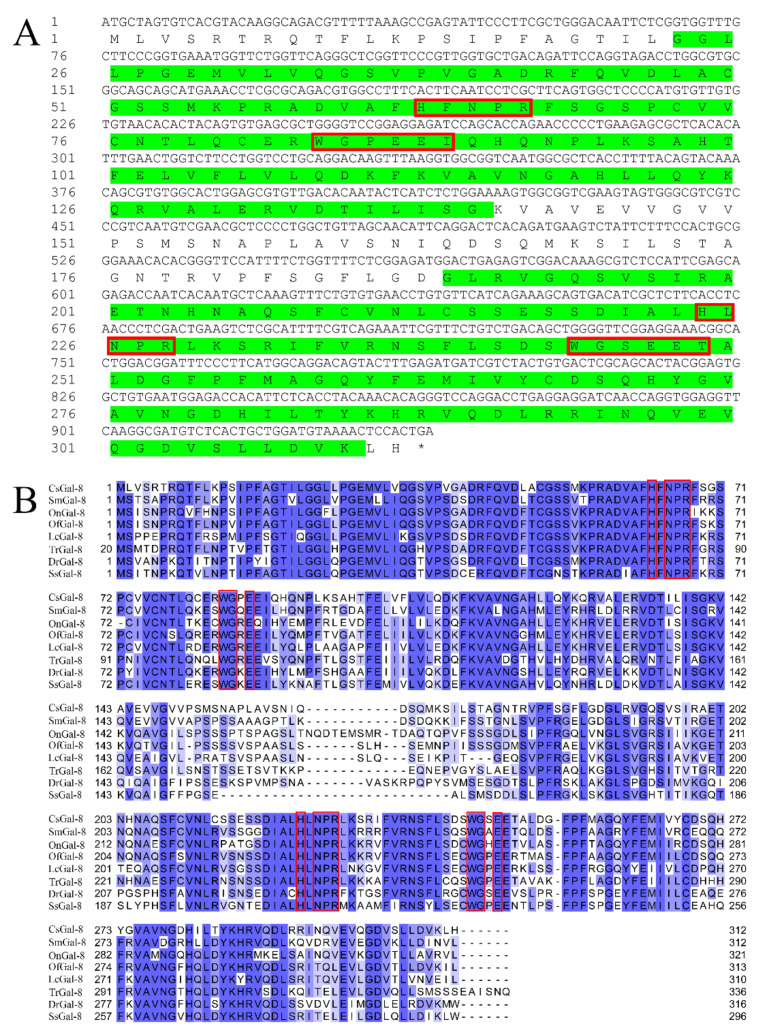
Sequence analysis of CsGal-8. (**A**) The cDNA sequence and deduced amino acid sequence of CsGal-8. The amino acid residues of the two predicted carbohydrate recognition domains (CRDs) are highlighted in green. Conserved motifs involved in carbohydrate binding are boxed in red. The stop codon is marked with an asterisk. The numbers along the left margin indicate the position of the nucleotides and amino acids. (**B**) Multiple sequence alignment of CsGal-8 with other teleost fish galectin-8 (Gal-8). Sm, *Scophthalmus maximus*; On, *Oreochromis niloticus*; Of, *Oplegnathus fasciatus*; Lc, *Larimichthys crocea*; Tr, *Takifugu rubripes*; Dr, *Danio rerio*; Ss, *Salmo salar*. The conserved motifs important for carbohydrate binding are indicated in red boxes. The similarity of the sequences is shown in blue, with darker shade indicating stronger similarity. GenBank accession numbers for protein sequences are as follows: SmGal-8, XP_035473203.1; OnGal-8, XP_003446682.2; OfGal-8, ANN46245.1; LcGal-8, TMS12152.1; TrGal-8, XP_003977516.2; DrGal-8, XP_003200715.1; SsGal-8, NP_001133778.1.

The open reading frame of CsGal-8 is 936 bp that encodes 312 amino acids (Figure 1A). CsGal-8 has an estimated molecular mass of 34.3 kDa and an isoelectric point of 7.14. Protein domain analysis indicated that CsGal-8 was a tandem repeat type galectin with two CRDs located at the N- and C-terminal regions. CsGal-8, especially the CRD domains, exhibits high sequence similarities (66–84%) with the Gal-8 of other teleost fish, i.e., *Danio rerio*, *Oreochromis niloticus*, *Salmo salar*, *Larimichthys crocea*, *Oplegnathus fasciatus*, *Scophthalmus maximus* and *Takifugu rubripes* (Figure 1B). The N-terminal CRD of CsGal-8 possesses the H-NPR and WG-EEI motifs, while the C-terminal CRD possesses the H-NPR and WG-EET motifs (Figure 1A). These motifs were predicted to be involved in carbohydrate binding. HXNPR and WGXE motifs are conserved in most other teleost Gal-8 (Figure 1B). The phylogenetic tree revealed that CsGal-8 was clustered together with the Gal-8 of *Scophthalmus maximus* (Figure S1). Three-dimensional structure modeling based on the crystal structure of human Gal-8 showed that the CRDs of CsGal-8 adopted compact globular structures formed by antiparallel β-sheet bundles (Figure S2).

### 2.2. Tissue Expression Profiles of CsGal-8 mRNA

**Figure 2:**
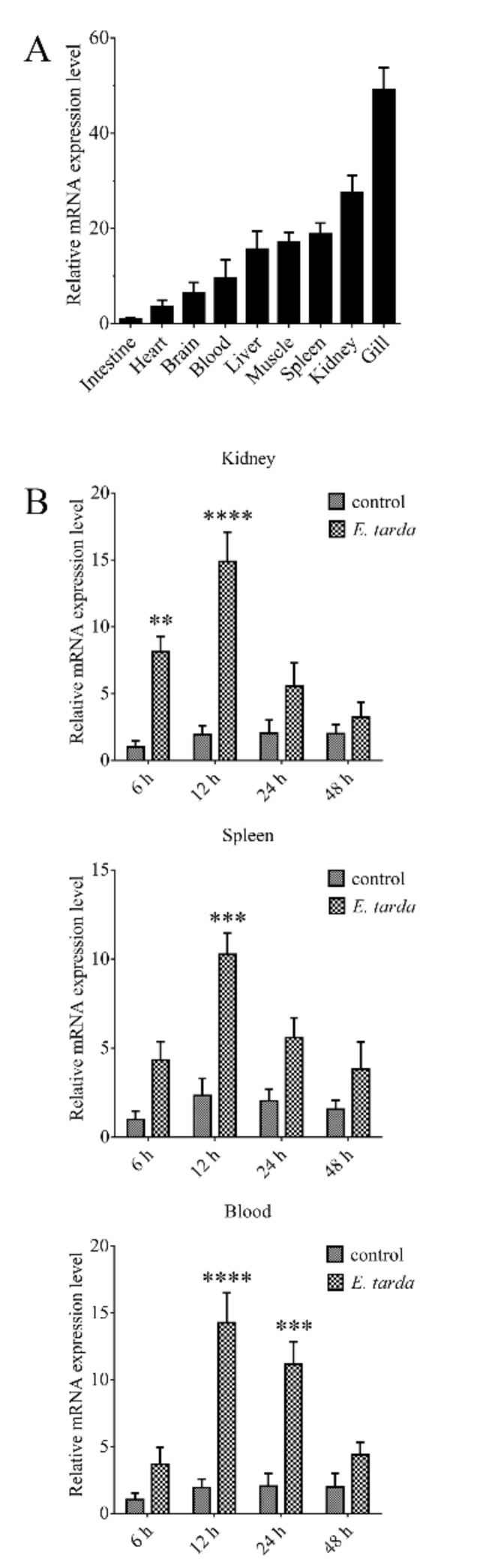
The expression of CsGal-8 in tongue sole tissues with and without bacterial infection. (**A**) CsGal-8 mRNA expression in the gill, kidney, spleen, muscle, liver, blood, brain and heart of tongue sole was determined by real-time-quantitative PCR (RT-qPCR). The expression levels are presented relative to that in intestine. (**B**) Tongue sole were infected with or without (control) *Edwardsiella tarda* for different hours, and the expression of CsGal-8 in blood, kidney and spleen was determined by RT-qPCR. Values are the means of three experimental replicates and shown as means ± S.D. ** *p* < 0.01, *** *p* < 0.001, **** *p* < 0.0001.

RT-qPCR showed that CsGal-8 mRNA was expressed at high levels in the gill, kidney, spleen, muscle and liver, and at lower levels in brain, heart and intestine (Figure 2A). Compared with the mRNA level in intestine, the mRNA levels in gill, kidney, spleen, muscle, liver, blood, brain and heart were 49.1-, 27.6-, 18.8-, 17.0-, 15.6-, 9.5-, 6.4- and 3.6-fold higher, respectively (Figure 2A). In tongue sole challenged with *E. tarda*, the expression of CsGal-8 in kidney increased significantly at 6 hpi (8.1-fold), peaked at 12 hpi (14.9-fold), and then fell back to normal level at 24 and 48 hpi (Figure 2B). In spleen, CsGal-8 expression increased significantly only at 12 hpi (10.3-fold) (Figure 2B). In blood, CsGal-8 expression was significantly enhanced at 12 hpi (14.3-fold) and 24 hpi (11.2-fold), and then fell back to normal level at 48 hpi (Figure 2B).

### 2.3. Subcellular Distribution and Extracellular Secretion of CsGal-8

**Figure 3:**
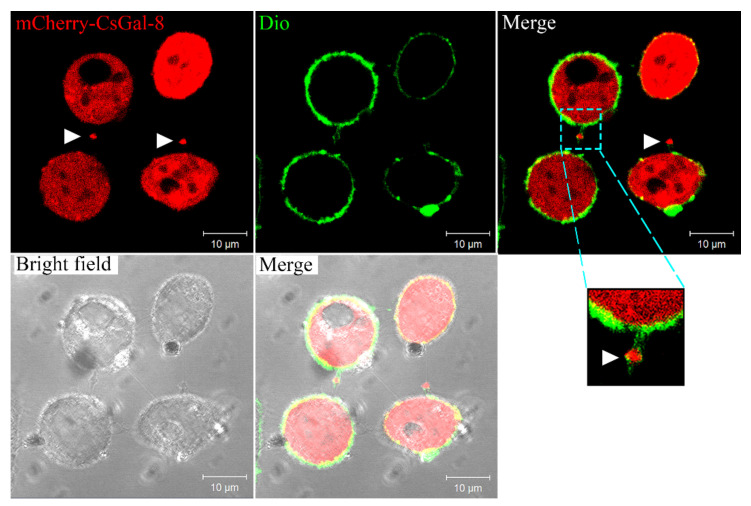
Intracellular distribution of CsGal-8. mCherry-tagged CsGal-8 was expressed in HeLa cells and observed with a confocal microscope. mCherry-tagged CsGal-8 was in red color and cell membrane was in green color after staining with DiO. Extracellular vesicles containing mCherry-tagged CsGal-8 are indicated by arrowheads.

Confocal microscopy showed that in HeLa cells expressing mCherry-tagged CsGal-8, CsGal-8 was observed to be diffused throughout in the cytoplasm (Figure 3). Furthermore, microvesicles containing CsGal-8 were captured releasing from the cell into the extracellular milieu (Figure 3). 

### 2.4. Carbohydrate Binding Specificity of Recombinant CsGal-8 (rCsGal-8)

**Figure 4:**
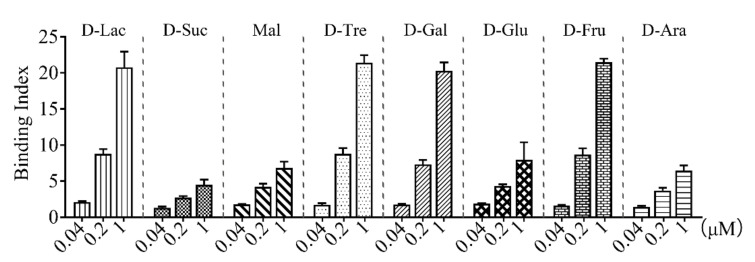
Carbohydrate binding ability of rCsGal-8. Binding of different concentrations of rCsGal-8 to various carbohydrates, including D-lactose (D-Lac), D-sucrose (D-Suc), maltose (Mal), D-trehalose (D-Tre), D-galactose (D-Gal), D-glucose (D-Glu), D-fructose (D-Fru) and D-arabinose (D-Ara), was detected by ELISA with PBS as a control. Values are the means of three experimental replicates and shown as means ± S.D.

rCsGal-8 (Figure S3) was purified from *E. coli* and examined for carbohydrate binding capacity. As shown in Figure 4, rCsGal-8 bound to different carbohydrates in a dose-dependent manner. rCsGal-8 exhibited relatively high binding activities towards lactose, trehalose, galactose, and fructose, and low binding activities to sucrose, maltose, glucose, and arabinose (Figure 4).

### 2.5. Bacteria Binding Ability of rCsGal-8

**Figure 5:**
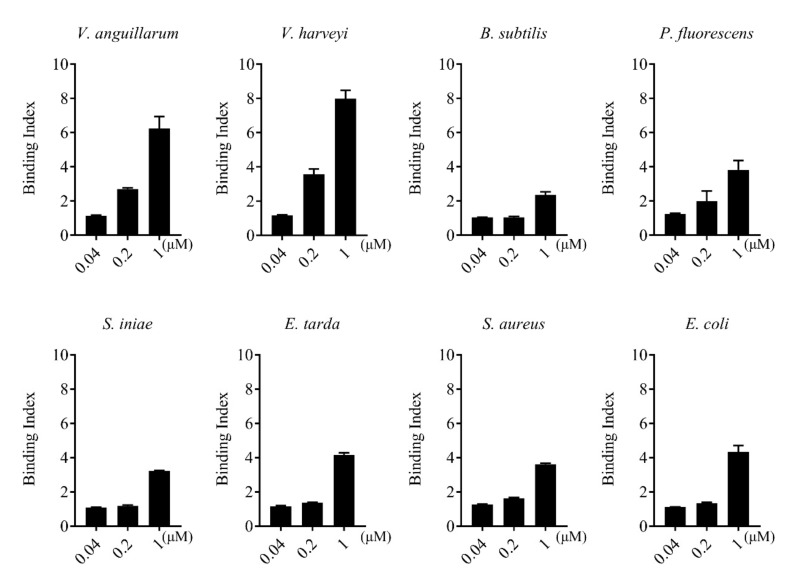
Bacteria binding ability of rCsGal-8. The binding of different concentrations of rCsGal-8 to different bacteria (*Vibrio anguillarum*, *Vibrio harveyi*, *Edwardsiella tarda*, *Streptococcus iniae*, *Pseudomonas fluorescens*, *Staphylococcus aureus*, *Escherichia coli*, and *Bacillus subtilis*) was determined by ELISA with PBS as a control. Values are the means of three experimental replicates and shown as means ± S.D.

**Figure 6:**
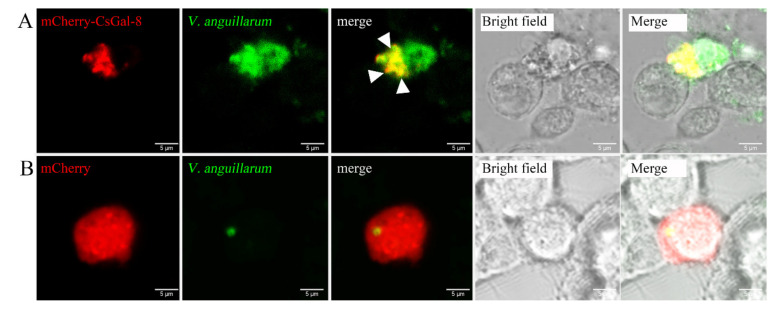
Co-localization of CsGal-8 with *Vibrio anguillarum*. RAW264.7 cells transfected with the vector expressing mCherry-tagged CsGal-8 (**A**) or the backbone vector (**B**) were incubated with FITC-labeled *V. anguillarum* for 2 h. The cells were then observed with a confocal microscope. Co-localization of CsGal-8 and *V. anguillarum* is indicated by arrowheads.

**Figure 7:**
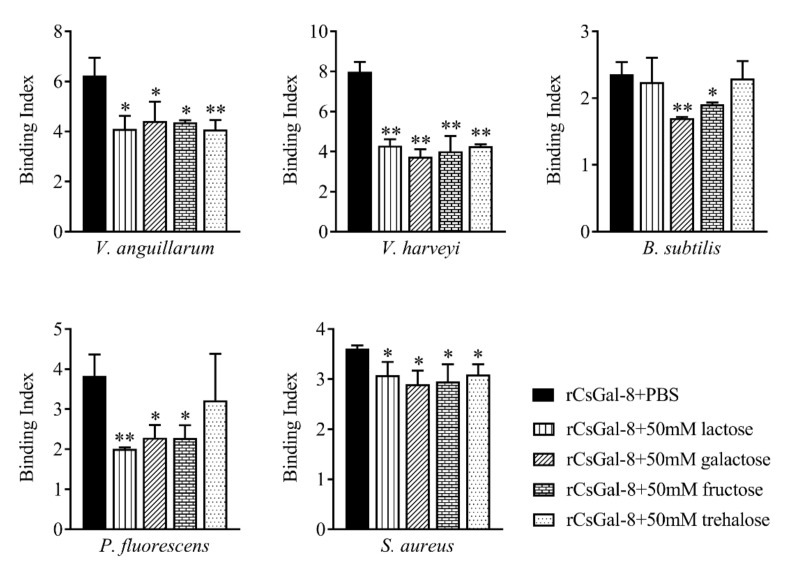
Inhibition effects of different carbohydrates on rCsGal-8 binding to bacteria. rCsGal-8 was pre-incubated with lactose, galactose, fructose, trehalose, or PBS (control), and the binding to different bacteria (*Vibrio anguillarum*, *Vibrio harveyi*, *Pseudomonas fluorescens*, *Staphylococcus aureus*, and *Bacillus subtilis*) was determined by ELISA. Values are the means of three experimental replicates and shown as means ± S.D. * *p* < 0.05, ** *p* < 0.01.

rCsGal-8 showed binding activity to various bacteria in a dose-dependent manner. At 0.2 µM, apparent binding was observed only with *V. anguillarum* and *V. harveyi*, while at 1 µM, binding was observed with *E. tarda*, *S. iniae*, *P. fluorescens*, *S. aureus*, *E. coli*, and *B. subtilis* as well (Figure 5). Microscopy detected co-localization of mCherry-tagged CsGal-8 with *V. anguillarum* in RAW264.7 cells (Figure 6). When pre-incubated with lactose, galactose, fructose, and trehalose, the binding ability of rCsGal-8 to *V. anguillarum*, *V. harveyi*, and *S. aureus* significantly decreased (Figure 7). The binding activity of rCsGal-8 to *P. fluorescens* was significantly inhibited by pre-incubation with lactose, galactose, and fructose, while the binding activity of rCsGal-8 to *B. subtilis* was significantly inhibited by pre-incubation with galactose and fructose (Figure 7). The binding activity of rCsGal-8 to *E. coli* was not inhibited by any of the above carbohydrates (data not shown).

### 2.6. rCsGal-8 Exhibits Antibacterial Effects

**Figure 8:**
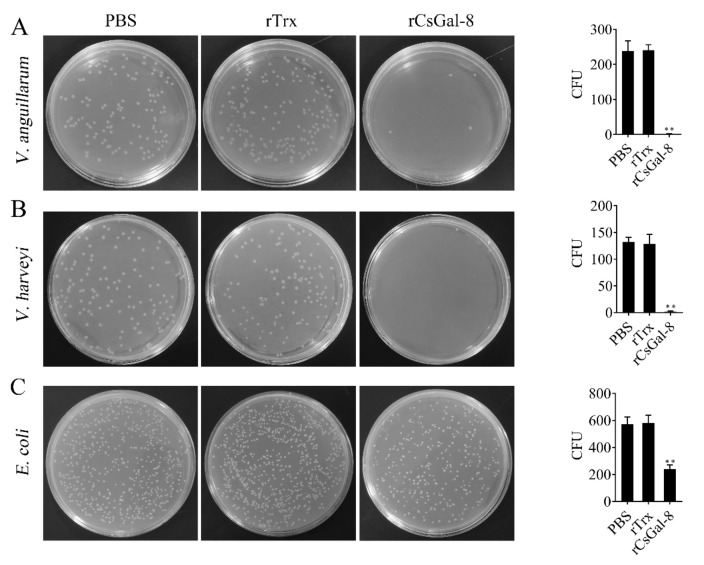
The bactericidal activity of rCsGal-8. *V. anguillarum* (**A**), *V. harveyi* (**B**) and *E. coli* (**C**) were incubated with 1 μM rCsGal-8 or rTrx or PBS (control) for 1 h. The bacteria were plated on LB plates and incubated for 12 h. The number of colony-forming unit (CFU) was counted and shown on the right panels. Values are the means of three experimental replicates and shown as means ± S.D. ** *p* < 0.01.

**Figure 9:**
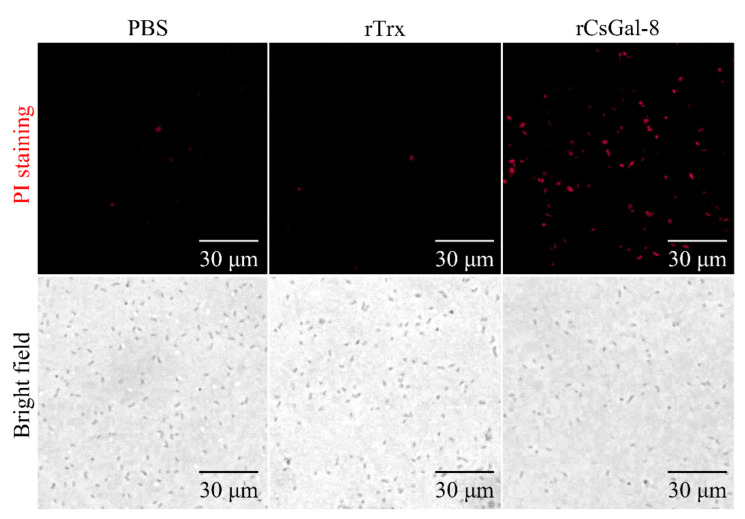
Propidium iodide staining analysis of bactericidal activity of rCsGal-8. *Vibrio anguillarum* were incubated with 1 μM rCsGal-8 or rTrx or PBS (control) for 30 min. Following the 30 min incubation, bacteria were stained with propidium iodide and observed under a fluorescence microscope.

**Figure 10:**
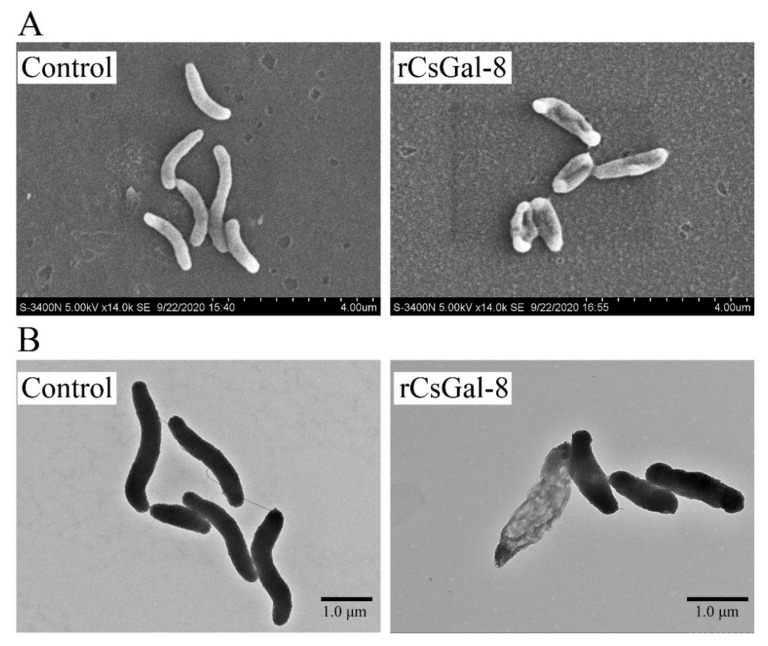
Ultra-structural analysis of rCsGal-8-treated *Vibrio anguillarum*. *V. anguillarum* was incubated with 1 μM rCsGal-8 or PBS (control) for 30 min and then examined with scanning electron microscopy (**A**) and transmission electron microscopy (**B**).

After treating with rCsGal-8, the viability of *V. anguillarum*, *V. harveyi*, and *E. coli* decreased by 90%, 90%, and 50%, respectively, whereas the viability of *S. aureus* and *S. iniae* was not affected (Figure 8 and data not shown). In contrast to rCsGal-8, treatment of *V. anguillarum* with the N- and C-terminal CRDs of CsGal-8 (designated rCsGal-8-N and rCsGal-8-C, respectively) failed to have any effect on the survival of *V. anguillarum* (Figure S4). Propidium iodide (PI) staining showed that after incubation with rCsGal-8, the cells of *V. anguillarum* became markedly PI-positive (Figure 9). SEM and TEM revealed that compared with control cells, *V. anguillarum* treated with rCsGal-8 was severely shrunken in morphology and exhibited deformed cellular structure (Figure 10). 

## 3. Discussion

Galectins are β-galactoside binding proteins with highly conserved CRDs. In mammals, the sequence identities among the CRDs of the same galectin from different species are 80–90% [1,5]. Galectins from different species of fish have also been reported to share high similarities, which range from ~40% to 90% [20,21,22]. Conserved sequence motifs, i.e., HXNPR and WGXEE, are located in the CRDs of most galectins. HXNPR and WGXEE are known to be involved in carbohydrate binding. Substitution of the Arg of HXNPR to His abolished the sugar-binding activity of human galectin-8, while substitution of the Arg of HXNPR to Asp resulted in the loss of lactose-binding ability of human galectin-9 [23,24,25]. In our study, we found that, like human Gal-8, the N-terminal CRD of CsGal-8 has the HFNPR and WGXEEI motifs. In contrast, the C-terminal CRD of CsGal-8 has the HLNPR and WGXEET motifs, which differ from that of human Gal-8 (HLNPR and WGXEER). These observations indicate that although the carbohydrate-interacting motifs are generally conserved in tongue sole, amino acid differences within the conserved motifs exist between tongue sole and humans, suggesting a potentially different carbohydrate preference between fish and humans. Sequence analysis showed that CsGal-8 lacks a classical signal peptide. However, CsGal-8 protein was found not only in the cytoplasm but also in the secreted micro-vesicles, which is in line with previous reports that galectins can be secreted through noncanonical pathways [6,26,27].

In fish, galectins have been found expressed in a variety of tissues and cell types. Gal-9 from large yellow croaker was abundantly expressed in skin, gill, heart, liver, kidney, spleen and intestine [14]. Congerin, a galectin of conger eel (*Conger myriaster*), was detected in gill, esophagus, skin and peritoneal cells [28]. Gal-3 of Nile tilapia (*Oreochromis niloticus*) was highly expressed in skin, gill, kidney and spleen [29]. In tongue sole, we found that CsGal-8 was constitutively expressed in multiple tissues at varied levels. CsGal-8 was highly expressed in immune associated tissues including gill, kidney, spleen, liver, and blood. Kidney and spleen are important immune organs of fish, and gill is the first line of immune defense [30,31,32,33]. These results suggest that CsGal-8 likely participates in the immune process of tongue sole. Consistently, *E. tarda* challenge significantly up-regulated CsGal-8 expression in kidney, spleen, and blood, which further supports an involvement of CsGal-8 in host immune defense.

Galectins are known to function as pattern recognition receptors through binding glycans on the surface of bacteria, fungi, parasites and viruses [9]. In mammals, human Gal-3 was shown to bind *Cryptococcus neoformans* and *Paracoccidioides brasiliensis* [34,35]. Mouse Gal-1 bound directly influenza virus through glycoproteins on the virus envelope, while mouse Gal-3 recognized *Candida albicans* by binding β-1,2-oligomannoside [36,37]. In fish, several galectins were reported to bind and agglutinate microbes. Gal-9 from *Carassius auratus* exhibited binding and agglutinating ability toward gram-positive and gram-negative bacteria and fungus [38], Gal-2 from rock bream bound and agglutinated various bacteria and a ciliate parasite [39]. The bacteria binding ability of Gal-3 from Nile tilapia was inhibited by LPS and lipoteichoic acid [40]. Similarly, our study showed that rCsGal-8 was able to bind a wide range of bacteria with different affinities. CsGal-8 was detected to be co-localized with *V. anguillarum* in RAW264.7 cells, suggesting that CsGal-8 was likely capable of bacteria binding inside phagocytes. Although galectins are generally considered to have high affinity for galactose or glycans containing beta-galactoside structure(s), human galectin-10 has been shown to bind mannose but have no affinity toward β-galactosides, due to its specific structure and the conformation of carbohydrate [4,41]. In line with these observations, in our study, competition assay showed that lactose, fructose, galactose and trehalose effectively suppressed the interaction between rCsGal-8 and bacteria, suggesting a broad carbohydrate binding capacity of rCsGal-8. However, it is of note that some sugars exhibited different degrees of inhibitory effect on the binding of rCsGal-8 to certain species of bacteria. As shown in Figure 7, compared to lactose, galactose exhibited a stronger inhibitory effect on the binding between *B. subtili*s and rCsGal-8, suggesting that the surface carbohydrate structure of *B. subtili*s involved in rCsGal-8 interaction is likely unique in a way that in which galactose plays a more important role than lactose in rCsGal-8 binding. These results suggested that CsGal-8 probably binds to different bacteria through different glycoconjugates on the surface of the bacterial cells. The binding ability of CsGal-8 to eukaryotic cells was not determined in this study. Given the carbohydrate binding capacity of rCsGal-8, it is possible that CsGal-8 may bind to the surface glycans of eukaryotic cells such as HeLa cells. 

In humans, galectins have been reported to kill pathogens directly. Gal-2 was bactericidal to *Helicobacter pylori* [42], while Gal-3 changed the morphology and induced death of *H. pylori* [43]. Gal-4 and Gal-8 could specifically kill blood group B positive *E. coli* by disrupting membrane integrity [44]. In fish, Gal-1 from rock bream and flounder showed protective activity against viral infection [12,45]. However, no reports of fish galectins with direct pathogen killing ability have been documented. In this study, we found that rCsGal-8 displayed potent bactericidal ability toward gram-negative bacteria but not gram-positive bacteria. With *V. anguillarum* and *V. harveyi* as targets, rCsGal-8 caused almost 100% killing. Compared with *V. anguillarum*, rCsGal-8 exhibited lower bactericidal activity against *E. coli*, which is in agreement with the observation that the binding affinity of rCsGal-8 to *V. anguillarum* was higher than that to *E. coli*. Thus, the bacterial killing ability of CsGal-8 is likely influenced by its bacterial binding capacity. PI staining indicated that after treatment with rCsGal-8, *V. anguillarum* was damaged in membrane, which enabled PI passage into the bacterial cells. Consistently, both scanning and transmission electron microscopy showed severe morphological changes in rCsGal-8-treated *V. anguillarum*. 

In conclusion, this study demonstrated that CsGal-8 is a broad range bacteria-binding lectin and can kill some gram-negative bacterial pathogens directly by inducing cellular membrane destruction. With its selected bactericidal activity, CsGal-8 may be used as an antimicrobial agent against specific bacterial pathogens.

## 4. Materials and Methods 

### 4.1. Animals and Ethic Statement

Healthy tongue sole *Cynoglossus semilaevis* (average 16.3 g) were purchased from a commercial farm. The fish were maintained at 20 °C in aerated seawater and acclimatized in the laboratory for 2 weeks prior to the study. The experiments involving live fish were approved by the Ethics Committee of Institute of Oceanology, Chinese Academy of Sciences (permit No. MB1506). 

### 4.2. Bacterial Strains and Culture Conditions

*Vibrio anguillarum*, *Vibrio harveyi*, *Edwardsiella tarda*, *Pseudomonas fluorescens*, and *Bacillus subtilis* were inoculated into Luria-Bertani (LB) broth and grew overnight at 28 °C. *Staphylococcus aureus* and *Escherichia coli* were inoculated into LB broth and grew overnight at 37 °C. *Streptococcus iniae* were inoculated to tryptic soy broth (TSB) and grew overnight at 28 °C. All overnight bacterial cultures were diluted 1:100 in fresh medium and grew to log phase. The bacteria were harvested by centrifugation and washed three times with sterile PBS.

### 4.3. Sequence Analysis

The BLAST algorithm was used to analyze the amino acid sequence of CsGal-8. Molecular weight and theoretical pI were calculated with the ProtParam tool at Expert Protein Analysis System (ExPASy). Three-dimensional structure was modeled using the SWISS-MODEL prediction algorithm. Multiple sequence alignment was carried out with ClustalW Multiple Alignment program. Simple modular architecture research tool (SMART) was performed to detect protein domains. Phylogenetic analysis was performed using the MEGA program.

### 4.4. Quantitative Real Time PCR (qRT-PCR)

To determine CsGal-8 expression in fish tissues under normal physiological conditions, tissue samples were isolated from the brain, gill, heart, kidney, liver, spleen, intestine, blood and muscle of healthy tongue sole after euthanization with tricaine methanesulfonate (Sigma, St. Louis, MO, USA). Total RNA was extracted and purified using TRIzol Reagent (Invitrogen, Carlsbad, CA, USA) according to the manufacturers’ instructions. The RNA was treated with DNase I to eliminate genomic DNA prior to cDNA synthesis. One microgram RNA was used to synthesize cDNA using the Superscript II Reverse Transcriptase (Invitrogen, Carlsbad, CA, USA). SYBR Green-based qRT-PCR was carried out with an Eppendorf Mastercycler (Eppendorf, Hamburg, Germany) as described previously [46]. The amplification conditions were as follows: 94 °C for 2 min, 40 cycles of 94 °C for 15 s, 55 °C for 15 s, and 57 °C for 30 s. Gene expression was calculated using comparative threshold cycle method (2−∆∆Ct) and normalized to that of β-actin [47]. To determine CsGal-8 expression during bacterial infection, bacterial challenge was performed as described previously [46]. Briefly, *E. tarda* was grown in LB broth at 28 °C to mid-log phase and harvested by centrifugation. The bacteria were washed with sterile PBS for three times and adjusted to a final concentration of 1 × 10^6^ CFU/mL. Tongue sole were injected intraperitoneally with 50 µL of PBS containing 1 × 10^6^ CFU/mL bacteria or 50 µL PBS (control). At 6, 12, 24, and 48 h post infection (hpi), kidney, spleen and blood were collected. qRT-PCR analysis of CsGal-8 expression in the collected tissues was performed as described above. CsGal-8 expression in kidney and blood was normalized to that of β-actin, while CsGal-8 expression in spleen was normalized to that of ribosomal protein L18 (RPL18) [47]. The primers for CsGla-8, β-actin, and RPL18 are CsGal-8 F1/R1, β-actin F/R, and RPL18 F/R (Table S1), respectively. All qRT-PCR assays were performed in triplicate.

### 4.5. Expression and Purification of Recombinant Proteins

Expression of the recombinant CsGal-8 (rCsGal-8) containing an N-terminal 6×His-tag was performed using the pET-28a plasmid (Novagen, Darmstadt, Germany) and *Escherichia coli* Bl21 (DE3) (Transgen, Beijing, China). The open reading frame of CsGal-8 was amplified by PCR using primers CsGal-8 F2 and CsGal-8 R2 (Table S1). The PCR product was gel-purified using the EZNA Gel Extraction Kit (Omega Biotek, Doraville, GA, USA) and cloned into PMD19-T simple vector (Takara, Dalian, China). The CsGal-8 coding sequence was then PCR amplified from the recombinant PMD19-T vector with primers CsGal-8 F3 and CsGal-8 R3 (Table S1). The PCR product was ligated into the Nde I and Xho I restriction sites of pET-28a. The recombinant plasmid was introduced into *E. coli* BL21 (DE3) by transformation. The transformant was grown in LB medium at 37 °C until OD600 0.4-0.6. Isopropyl β-D-1-thiogalactopyranoside (1 mM) (AiKB, Qingdao, China) was added to the culture, followed by 4 h growth at 37 °C. rCsGal-8 was purified using Ni-NTA Agarose (QIAGEN, Germany) according to the manufacturers’ instructions. The purified rCsGal-8 was refolded in refolding buffer and dialyzed against PBS as reported previously [48]. Recombinant C- and N-terminal CRDs of CsGal-8 (designated rCsGla-8-C and rCsGla-8-N, respectively) were expressed and purified similarly using pET-28a and Bl21 (DE3). Briefly, the open reading frames of the C- and N-terminal CRD of CsGal-8 were amplified and ligated into the Nde I and Xho I restriction sites of pET-28a. Expression and purification procedures were performed as described above. The primers for CsGla-8-N and CsGla-8-C are CsGla-8-N F/R and CsGla-8-C F/R (Table S1), respectively. To obtain recombinant thioredoxin (rTrx), pET-32a plasmid, which expresses His-tagged Trx, was introduced into *E. coli* BL21 (DE3) by transformation. Expression and purification of rTrx were performed similarly as above for rCsGal-8. The recombinant proteins were analyzed in 12% SDS-PAGE and stained with Coomassie Brilliant Blue R-250. Protein concentration was determined with an Enhanced BCA protein assay kit (Beyotime, Shanghai, China).

### 4.6. Carbohydrate and Bacteria Binding Assay

Carbohydrate binding was examined using enzyme-linked immunosorbent assay (ELISA) as described previously [49]. Briefly, lactose, sucrose, maltose, trehalose, galactose, glucose, fructose and arabinose were dissolved in coating buffer (0.159% Na2CO3, 0.293% NaHCO3, pH 9.6) and used to coat 96-well microtiter plates at 4 °C for 8 h. After coating, the plates were blocked with 3% BSA in PBS for 8 h at 4 °C. The plates were washed three times with PBS, and 100 μL rCsGal-8 at different concentrations (0.04 μM, 0.2 μM and 1 μM) was added to each well. The plates were incubated at room temperature for 2h, followed by washing three times with PBST (0.1 M PBS,0.1% Tween-20). Horseradish peroxidase (HRP)-labeled mouse monoclonal anti-His antibody (ABclonal, Hubei, China) was added to the plates, and the plates were incubated at room temperature for 1h. The plates were washed five times with PBST, and 100 μl substrate solution (3, 3′,5, 5′-Tetramethylbenzidine, (Beyotime, Shanghai, China) was added to each well of the plates. The plates were incubated at 37 °C for 10min, and 50 μL ELISA stop solution (Solarbio, Beijing, China) was added to each well to stop the reaction. The absorbance at 450 nm was measured using a Precision microplate reader (Molecular Devices, Toronto, Canada). PBS was used as a control in the assay. Binding index = A450 of protein/A450 of PBS. 

For bacterial binding, *V. anguillarum*, *V. harveyi*, *E. tarda*, *S. iniae*, *P. fluorescens*, *S. aureus*, *E. coli*, and *B. subtilis* were resuspended in coating buffer to a final concentration of 1 × 10^8^ CFU/mL and used to coat 96-well microtiter plates at 4 °C for 8h. The binding of the bacteria to rCsGal-8 was performed with ELISA as described above. To examine the inhibitory effects of carbohydrates on the binding of rCsGal-8 to bacteria, rCsGal-8 was pre-incubated with 50 mM lactose, trehalose, galactose, or glucose for 30 min before adding to the bacteria coated 96-well microtiter plates, and rCsGal-8 binding to bacteria was determined as above with ELISA.

### 4.7. Confocal Microscopy

To examine the subcellular distribution of CsGal-8, the CsGal-8 coding sequence was PCR amplified with primers CsGal-8 F4 and R4 (Table S1), and the PCR product was ligated into the NheI and XhoI restriction sites of pmCherry-N1 expression vector (Clontech, Mountain View, CA, USA), resulting pmCherry-N1-CsGal-8. HeLa cells were cultured in Dulbecco’s minimal Eagle’s medium (DMEM) (Gibco, Carlsbad, CA, USA) supplemented with 10% fetal bovine serum (FBS) (Gibco, Carlsbad, CA, USA) at 37 °C in a 5% CO_2_ incubator. For transfection, HeLa cells were seeded onto glass-bottom culture dishes (Nest Scientific, Rahway, NJ, USA) and cultured to a confluency of 70–80%. The cells were transfected with pmCherry-N1-CsGal-8 using PolyJet in vitro DNA transfection reagent (SignaGen Laboratories, Ijamsville, MD, USA) for 24 h. The cells were then observed with a Carl Zeiss LSM 710 confocal microscope (Carl Zeiss, Jena, Germany). 

To examine the subcellular localization of CsGal-8 and *V. anguillarum*, RAW 264.7 cells were cultured and transfected with pmCherry-N1-CsGal-8 or the backbone vector (control) as above. At 24 h post transfection, the cells were incubated with FITC-labeled *V. anguillarum*. After 2 h incubation, the cells were observed with the confocal microscope described above.

### 4.8. Bacterial Killing Assay

Bacteria were grown to mid-log phase as described above. The bacterial cells were harvested by centrifugation and washed with PBS. The bacteria were adjusted to 1 × 10^6^ CFU/mL in PBS. rCsGal-8 or rTrx was added to the bacterial suspension at the final concentration of 1 μM. For the control sample, PBS was added to the bacterial suspension. The mixture was incubated at room temperature for 1 h and then plated on LB or TSB agar plates as described above. After incubation for 12 h, colony forming units (CFUs) were counted. The bacterial killing activity of rCsGal-8N and rCsGal-8C was determined in the same way.

### 4.9. Electron Microscopy

*V. anguillarum* was grown to mid-log phase and harvested by centrifugation. The bacteria were washed three times with PBS and adjusted to 1 × 10^6^ CFU/mL in PBS. rCsGal-8 was added to the bacterial suspension at a final concentration of 1 μM and incubated at room temperature for 30 min. For the control group, the bacteria were incubated with PBS. After incubation, the bacteria were centrifuged and fixed at 4 °C overnight with 2.5% glutaraldehyde in PBS, followed by washing three times with PBS. The bacteria were dehydrated through an increasing concentration of ethanol (30%, 50%, 70%, 80%, 90% and 100%). The bacteria were resuspended in isopentyl acetate and observed with a scanning electron microscope (SEM) (Hitachi, S-3400N, Tokyo, Japan) and a transmission electron microscope (TEM) (Hitachi, HT7700, Japan).

### 4.10. Propidium Iodide (PI) Staining

*V. anguillarum* was grown to mid-log phase and harvested by centrifugation. The bacteria were washed three times with PBS and adjusted to 1 × 10^6^ CFU/mL in PBS. rCsGal-8 or rTrx was added to the bacterial suspension at a final concentration of 1 μM and incubated at room temperature for 30 min. For the control group, the bacteria were incubated with PBS. After incubation, PI was added to the mixture at a final concentration of 2 μg/mL. The mixture was incubated in the dark at room temperature for 10 min. The bacteria were visualized with an Olympus DP70 microscope (Japan).

### 4.11. Statistical Analysis

Statistical analysis was performed with the data of triplicate experiments. Data were analyzed using the two-sample Student’s t test and expressed as the mean ± SD. Significance was defined as *p* < 0.05.

## Data Availability

Data is contained within the article or Supplementary Material.

## Supplementary Materials

The following are available online at https://www.mdpi.com/1422-0067/22/1/376/s1.
