# Supplementary material for: A Fish Galectin-8 Possesses Direct Bactericidal Activity
Source: Int J Mol Sci. 2020 Dec 31;22(1):376. doi: 10.3390/ijms22010376 (PMC7796122; doi:10.3390/ijms22010376)

Supplementary data

Table S1. Primers used in the present study.

Primer	Sequence (5'-3')
CsGal-8 F1	AGCAGTGACATCGCTCTTCACC
CsGal-8 R1	ATTCACAGCCACTCCGTAGTGC
β-actin F	GCACGGTATTGTGACCAACTGG
β-actin R	CAGGGGAGCCTCTGTGAGC
RPL18 F	GAACCCTACCCCTCCTCTGT
RPL18 R	TACGAGAGTCGTAACGCAGC
CsGal-8 F2	ATGCTAGTGTCACGTACAAGGCAG
CsGal-8 R2	TCAGTGGAGTTTTACATCCAGCAGTG
CsGal-8 F3	GGAATTCCATATGCTAGTGTCACGTACAAGGCAG
CsGal-8 R3	CCGCTCGAGTCAGTGGAGTTTTACATCCAGCAGTG
CsGal-8-N F	GGAATTCCATATGATGGGTGGTTTGCTTCCC
CsGal-8-N R	CCGCTCGAGTCCAGAGATGAGTATTGTGTCAACA
CsGal-8-C F	GGAATTCCATATGATGGGACTGAGAGTCGGACA
CsGal-8-C R	CCGCTCGAGTTTTACATCCAGCAGTGAGACATC
CsGal-8 F4	CTAGCTAGCATGCTAGTGTCACGTACAAGGCAG
CsGal-8 R4	CCGCTCGAGGTGGAGTTTTACATCCAGCAGTGAGA

Figure S1. Phylogenetic analysis of CsGal-8. The phylogenetic tree was constructed with the neighbor-joining algorithm. CsGal-8 is marked with an asterisk.

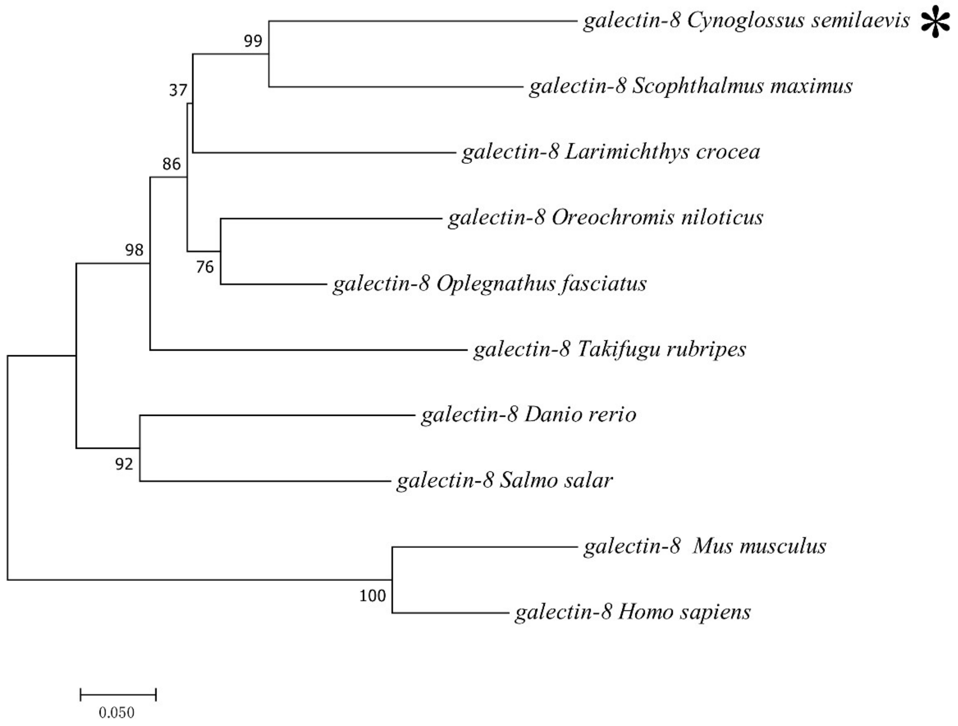

Figure S2. Three-dimensional structure of CsGal-8 modeled using the SWISS-MODEL prediction algorithm based on human Gal-8.

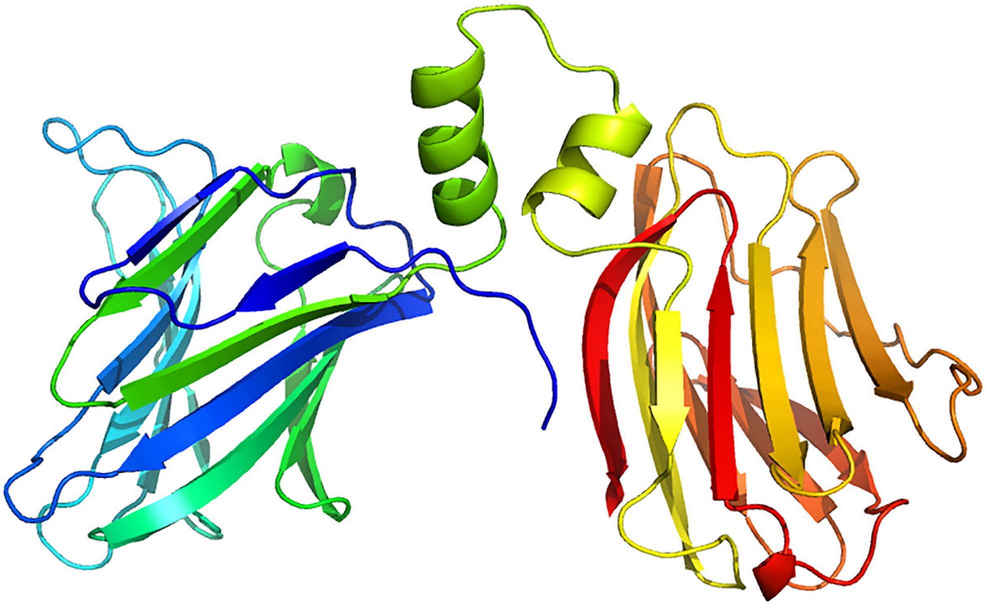

Figure S3. SDS-PAGE analysis of recombinant CsGal-8 (rCsGal-8). rCsGal-8 was expressed in *E. coli* and purified by Ni-NTA affinity chromatography. The total proteins, the Flow-through portion, and the purified rCsGal-8 were subjected to SDS-PAGE and stained with Coomassie brilliant blue R250 after electrophoresis.

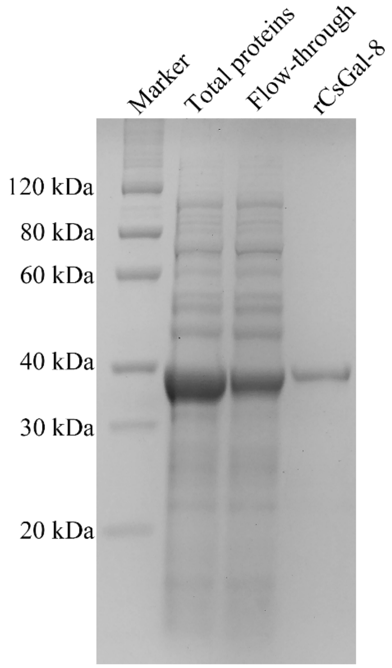

Figure S4. The effects of rCsGal-8-N and rCsGal-8-C on *Vibrio anguillarum* viability. *V. anguillarum* was incubated with or without (control) 1 μM rCsGal-8, rCsGal-8-N, or rCsGal-8-C for 1 h. The number of surviving bacteria was counted and shown as colony-forming unit (CFU). Values are the means of three experimental replicates and shown as means ± S.D.. ** $p<0.01$.

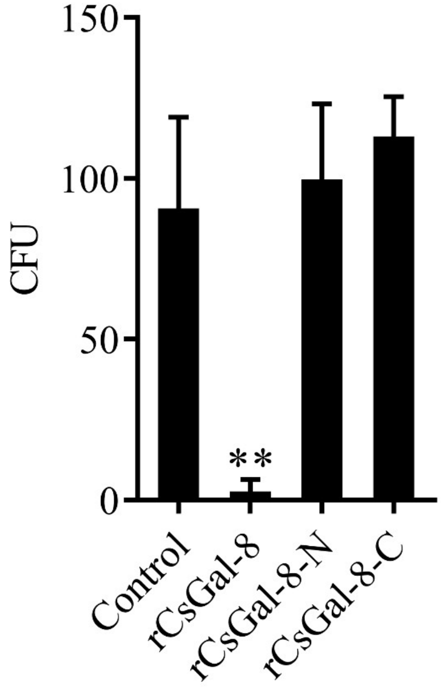

Supplement: Supplementary file 1 [file ijms-22-00376-s001.pdf]
